# Ubiquitylation of the acetyltransferase MOF in *Drosophila melanogaster*

**DOI:** 10.1371/journal.pone.0177408

**Published:** 2017-05-16

**Authors:** Sarah Schunter, Raffaella Villa, Victoria Flynn, Jan B. Heidelberger, Anne-Kathrin Classen, Petra Beli, Peter B. Becker

**Affiliations:** 1 Molecular Biology Division, Biomedical Center and Center for integrated Protein Science Ludwig-Maximilians-University, Munich, Germany; 2 Institute of Molecular Biology (IMB), Mainz, Germany; 3 Center for Biological Systems Analysis (ZSBA), Freiburg, Germany; Dana Farber Cancer Institute, UNITED STATES

## Abstract

The nuclear acetyltransferase MOF (KAT8 in mammals) is a subunit of at least two multi-component complexes involved in transcription regulation. In the context of complexes of the ‘Non-Specific-Lethal’ (NSL) type it controls transcription initiation of many nuclear housekeeping genes and of mitochondrial genes. While this function is conserved in metazoans, MOF has an additional, specific function in *Drosophila* in the context of dosage compensation. As a subunit of the male-specific-lethal dosage compensation complex (MSL-DCC) it contributes to the doubling of transcription output from the single male X chromosome by acetylating histone H4. Proper dosage compensation requires finely tuned levels of MSL-DCC and an appropriate distribution of MOF between the regulatory complexes. The amounts of DCC formed depends directly on the levels of the male-specific MSL2, which orchestrates the assembly of the DCC, including MOF recruitment. We found earlier that MSL2 is an E3 ligase that ubiquitylates most MSL proteins, including MOF, suggesting that ubiquitylation may contribute to a quality control of MOF’s overall levels and folding state as well as its partitioning between the complex entities. We now used mass spectrometry to map the lysines in MOF that are ubiquitylated by MSL2 in vitro and identified in vivo ubiquitylation sites of MOF in male and female cells. MSL2-specific ubiquitylation in vivo could not be traced due to the dominance of other, sex-independent ubiquitylation events and conceivably may be rare or transient. Expressing appropriately mutated MOF derivatives we assessed the importance of the ubiquitylated lysines for dosage compensation by monitoring DCC formation and X chromosome targeting in cultured cells, and by genetic complementation of the male-specific-lethal *mof*^*2*^ allele in flies. Our study provides a comprehensive analysis of MOF ubiquitylation as a reference for future studies.

## Introduction

The lysine acetyltransferase KAT8 (also MYST1) is a subunit of several large complexes that regulate transcription initiation and elongation. In *Drosophila*, KAT8 resides in two major regulatory complexes. In context of the ‘NSL’ (non-specific-lethal) complex, the enzyme associates with many housekeeping genes, where it acetylates histone H4 of promoter nucleosomes to facilitate transcription initiation [1–6]. In mammals a variant of the NSL complex also regulates the expression of respiratory genes in mitochondria, but in this case the substrate for its acetylation is unknown [7].

The name MOF (males-absent-on-the-first) reveals how the gene was originally discovered. MOF mutant flies show male-specific lethality due to their inability to compensate for the fact that male flies only have a single X chromosome, while females have two. Dosage compensation balances this sex chromosome monosomy in males by increasing the transcription of most genes on the X in the two-fold range [8]. MOF is crucial to this activation as it acetylates histone H4 at lysine 16 in transcribed chromatin, a modification known to loosen up the chromatin fiber. The known functional domains of MOF, featuring the histone acetyltransferase (HAT) and a chromobarrel domains (CBD), reside in the C-terminal half of the enzyme. Unlike its human orthologue, the *Drosophila* enzyme exhibits a large, unstructured and highly acidic (pI ~3.88) N-terminus of roughly 350 amino acids, which is required in the context of dosage compensation. The N-terminus has been shown to modulate the substrate binding properties of the HAT domain, possibly by conformational autoinhibition [9].

MOF is targeted to the X chromosome by its association with the male-specific-lethal dosage compensation complex (MSL-DCC). This complex consists of three MSL proteins (MSL1, MSL2 and MSL3), the RNA helicase MLE, two long, non-coding *roX* RNAs and MOF. The complex is targeted to the X chromosome through the male-specific MSL2 subunit, which recognizes specific MREs (MSL Recognition Elements) and PionX sites on the X chromosome [10–12], from which it reaches out to acetylate active chromatin in its vicinity [13].

The selective targeting of the X chromosome requires carefully adjusted levels of MSL-DCC. Experimental elevation of MSL-DCC levels leads to inappropriate binding to autosomal sites and unbalanced transcription. MSL2 is crucial to this quantitative regulation as it is the only subunit that is expressed exclusively in males. MSL2 expression is regulated at multiple levels [14–17] as its concentration in the nucleus determines how much of MOF and of the other MSL subunits are recruited into the MSL-DCC. Moreover, we recently found that MSL2 is involved in feedback regulation through an E3 ubiquitin ligase activity [18]. MSL2 ubiquitylates itself as well as MOF, MSL1 and MSL3. We speculated that this ubiquitylation may serve to install homeostatic control of tuned MSL-DCC levels [18]. However, we also detected chromatin-bound ubiquitylated MSL1 in steady-state conditions, giving rise to the speculation that certain ubiquitin marks may serve other functions besides targeting degradation. Ubiquitylation is not only involved in protein degradation but can also act as a signal that regulates molecular interactions [19–21].

In our earlier study, the pattern of MOF ubiquitylation appeared relatively simple with low levels of poly-ubiquitylated species [18], which encouraged the systematic characterization of MOF ubiquitylation presented here. We mapped the sites of MSL2-dependent ubiquitylation in vitro and determined the prevalent ubiquitylation sites in male and female cultured cells. Expressing mutated MOF proteins, in which modified lysines were mutated to arginine, we evaluated the importance of these residues for the MSL-DCC formation and X chromosome targeting in tissue culture cells as well as for their dosage compensation function in male flies.

## Results

### MSL2 ubiquitylates MOF in vitro

**Fig 1:**
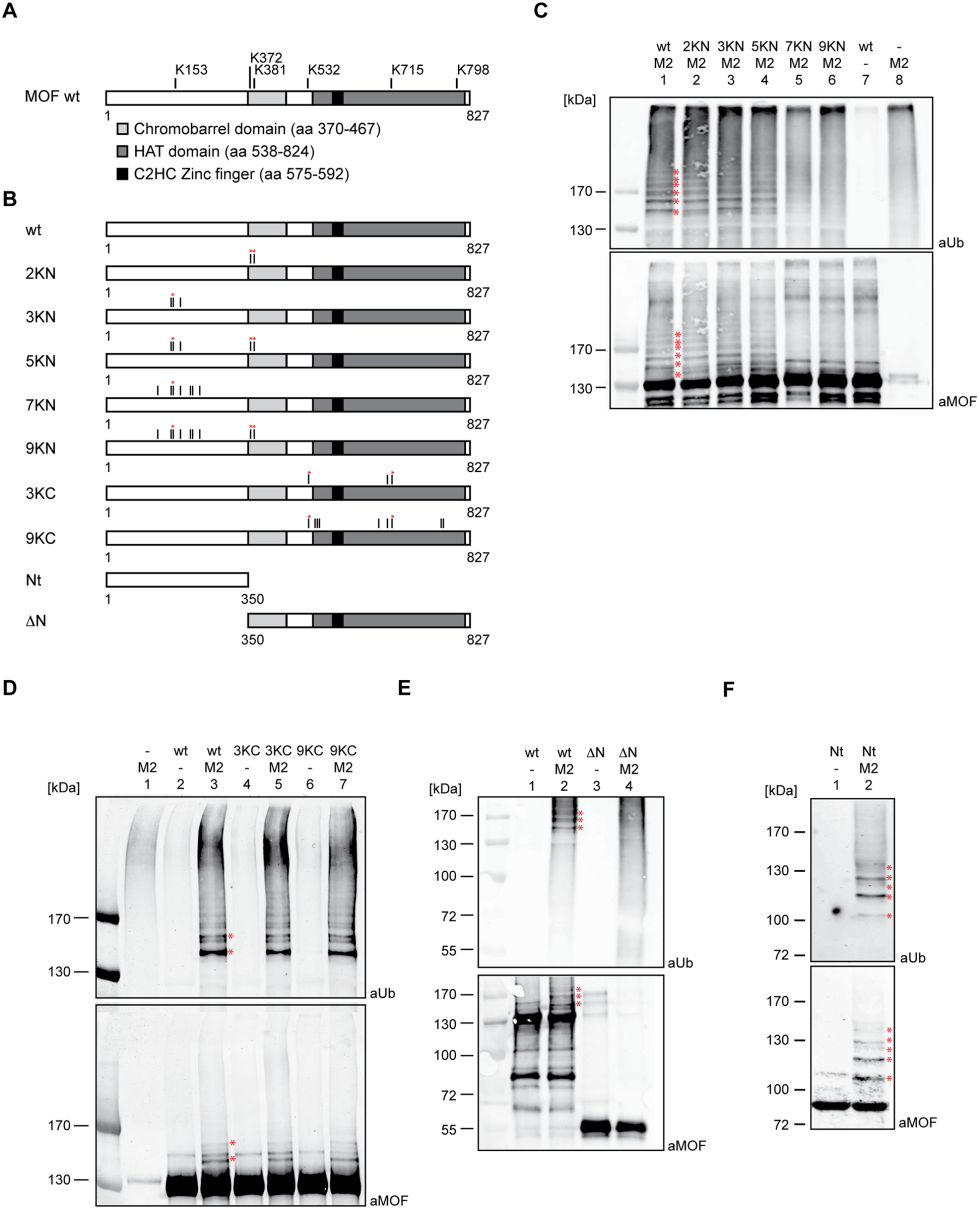
MSL2 ubiquitylates MOF *in vitro*. (A) Schematic representation of MOF protein and the ubiquitylated lysines identified by mass spectrometry in vitro. MOF contains an unstructured N-terminal region, a globular chromobarrel and a histone acetyltransferase domains (light and dark gray box, respectively). MOF comprises a C2HC Zinc finger within the histone acetyltransferase domain (black box). Lysines that are ubiquitylated by MSL2 are indicated. Bold bars mark ubiquitylation found in 3 out of 3 biological replicates, dashed bars point to ubiquitylation only detected in 1 out of 3 experiments. (B) Schematic representation of MOF mutants generated in this study. Black bars indicate K to R mutations. Red asterisks indicate the lysines that are ubiquitylated in vitro as in (A). (C) In vitro ubiquitylation of MOF wt and KN MOF mutants. Ubiquitylation assays contained recombinant E1 and E2 enzymes, his-ubiquitin and ATP. MSL2 and different MOF substrates were added as indicated. Ubiquitylated proteins were detected by Western blotting using antibodies specific for ubiquitin (aUb, top) and MOF (aMOF, bottom). In the absence of substrate protein MSL2 exhibits autoubiquitylation on itself as detected in lane 8. Protein size markers are indicated to the left (kDa). Red asterisks indicate bands that correspond to ubiquitylated forms of MOF. (D) In vitro ubiquitylation as in (C) with KC MOF mutant substrates. (E) In vitro ubiquitylation as in (C) with MOF ΔN substrate. (F) In vitro ubiquitylation in (C) with MOF-Nt substrate.

To identify the sites on MOF that are ubiquitylated by MSL2 in vitro, recombinant MOF was incubated with MSL2 in a ubiquitylation assay and subjected to mass spectrometry. Ubiquitylated peptides were identified by the characteristic mass shift resulting from the attachment of a di-glycine tag at the ubiquitylation site that remains from the C-terminus of ubiquitin after trypsin digestion. Ubiquitylated lysines were found in the unstructured N-terminus, at the edge of the chromobarrel domain and distributed throughout the C-terminal HAT domain at positions K153, K372, K381, K532, K715 and K789 (Fig 1A and S1 Fig). In addition to these sites, we also identified several neighboring ubiquitylation sites with lower localization probability or search engine score (S1 Table). MSL2 is apparently able to synthesize branched oligo-ubiquitin chains with different linkages since we identified specific peptides corresponding to ubiquitin linked at lysines K11, K48 and K63. To score the relative contribution of each site to overall MOF ubiquitylation we created MOF expression constructs bearing specific K to R point mutations (Fig 1B). The two modified lysines in the chromobarrel domain were mutated separately (construct ‘2KN’), as were the three lysines in the N terminus (‘3KN’). Construct ‘5KN’ combines these two sets of mutations. The three substrate lysines in the HAT domain were mutated in construct ‘3KC’. As ubiquitylation shows a high degree of promiscuity, we also generated constructs where additional lysines close to the modified ones were mutated. In construct ‘7KN’ all lysines in the unstructured N-terminus are converted to R. ‘9KN’ combines the mutations present in ‘2KN’ and ‘7KN’ and expresses a MOF derivative lacking all lysines present up to position 400. The C-terminal HAT domain contains many lysines, therefore we mutated just 9 lysines (‘9KC’) that are predicted to be surface-exposed by analogy to the HAT domain of human MOF, for which the structure is known [22]. In addition, we expressed the unstructured N-terminus alone (Nt) or a MOF derivatives lacking this unstructured part (ΔN; Fig 1B).

Equivalent amounts of recombinant wild type MOF (wt) and all mutant forms (S2 Fig) were tested for MSL2-dependent ubiquitylation in vitro. Western blot analyses identify ubiquitylated MOF in bands that are detected with antibodies against both, ubiquitin and MOF, in a dual-color infrared imaging system. MSL2 ubiquitylated MOF-wt and the 2KN, 3KN and 5KN proteins to the same extent, whereas ubiquitylation of the 7KN and 9KN mutants was dramatically reduced (Fig 1C). The residual signal detected in the ubiquitylation blot of these mutants was due to MSL2 auto-ubiquitylation (Fig 1C, lane 8). No ubiquitylation signal was detected in the absence of MSL2 (Fig 1C, lane 7). By contrast, even mutating 9 surface-exposed lysines in the C-terminal HAT domain had no effect on the overall amount of ubiquitylated MOF (Fig 1D), suggesting that the ubiquitylation pattern revealed by these experiments derives mainly from modification of the N-terminal half of MOF. This was confirmed by analyzing the deletion mutants: A MOF derivative lacking the MOF N-terminus was not ubiquitylated by MSL2 (Fig 1E), whereas MOF-Nt was readily ubiquitylated (Fig 1F). Taken together, these results suggest that MSL2 preferentially ubiquitylates MOF within the 400 N-terminal amino acids in vitro.

### MOF is ubiquitylated in vivo

**Fig 2:**
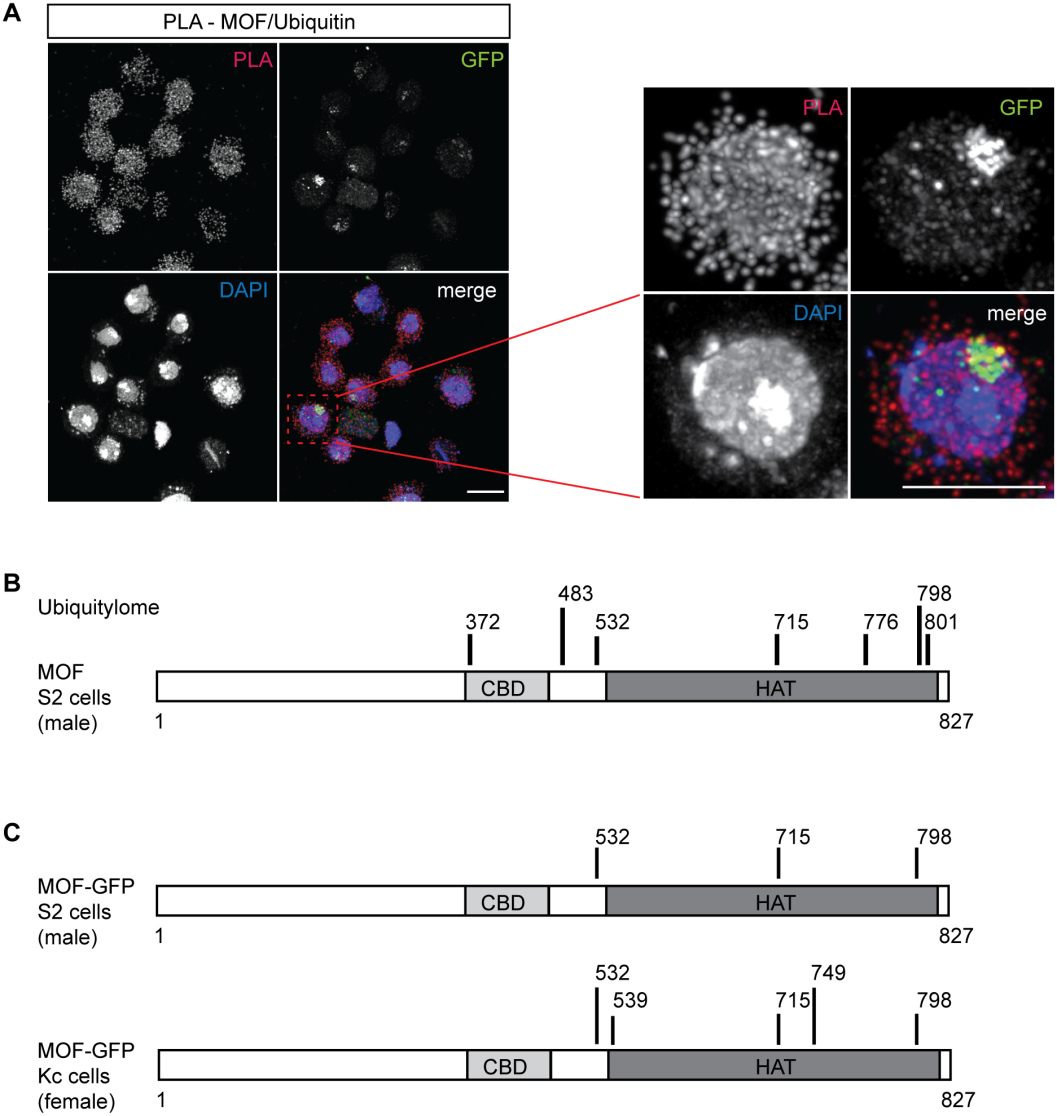
MOF is ubiquitylated in vivo. (A) Detection of MOF ubiquitylation in vivo using proximity ligation assays (PLA). Cell lines stably expressing MSL2-GFP were subjected to PLA assays with anti-MOF and anti-ubiquitin antibodies. The right panel shows an enlargement of one representative cell from the left panels. ‘Merge’ shows an overlay of the PLA signal (red, ubiquitylated MOF), the anti-GFP signal (enrichment of MSL2-GFP at the X chromosome territory, green) and counterstaining of DNA with DAPI (blue). Scale bars represent 10 μm. (B) The ubiquitylome of MOF in S2 cells. S2 protein extracts were digested with trypsin and ubiquitylated peptides were immunoprecipitated with a di-glycine antibody and identified by LC-MS/MS. A schematic summary of ubiquitylation sites are presented. Black bars indicate ubiquitylated lysines on MOF. CBD: chromobarrel domain, HAT: histone acetyltransferase domain. One biological replicate is shown. (C) Summary of ubiquitylation sites on overexpressed MOF-GFP. MOF-GFP was affinity-purified from stable S2 (male) and Kc (female) overexpression lines and ubiquitylated lysines (back bars) were identified by mass spectrometry. S2 cells were analyzed in four biological replicates, for Kc cells two replicates were performed.

MOF ubiquitylation may predispose for degradation and therefore be difficult to detect in vivo. We employed the highly sensitive and specific proximity ligation assay (PLA), which allow the detection of protein-protein interactions as well as protein modifications [23–26] to search for ubiquitylated forms of MOF in S2 cells (a clonal derivative selected for tetraploidy, see Materials and methods) stably expressing MSL2 fused to the green fluorescent protein (GFP) as marker for the X chromosome territory [18]. PLA yields a signal if two distinct epitopes bound by specific antibodies (e.g. MOF and ubiquitin) are within a 40 nm distance, with a good chance that the ubiquitylation is indeed on MOF as opposed to a different, close-by protein. Appropriate secondary antibodies are conjugated with complementary DNA probes that, if sufficiently close, anneal to form a primed template. During rolling circle amplification of this DNA fluorescently labeled nucleotides are incorporated and locally enriched. Fixed cells were stained with primary antibodies against MOF and ubiquitin, subjected to PLA and fluorescent foci were detected by fluorescent microscopy (Fig 2A and S3A Fig). Many interaction foci were detected in the cytoplasm and in the nuclei on autosomes and the X chromosome when antibodies directed against MOF and ubiquitin were included (Fig 2A). No signal was scored if the MOF antibody was omitted or if MOF was depleted from the cells by RNA interference (S3A Fig). We conclude that MOF is ubiquitylated in vivo. Although some PLA signal is detected on the X chromosomal territory, the picture is dominated by signal on autosomes, possibly due to MOF residing in the NSL complex [2,5,6] and in the cytoplasm, likely corresponding to mitochondrial MOF [27].

While this approach demonstrates the existence of MOF ubiquitylation in vivo in the absence of proteasome inhibitor, it does not reveal the modified residues. To identify ubiquitylated lysines in vivo we immunoprecipitated di-glycine-containing peptides with a specific antibody from *Drosophila* S2 cell lysates expressing endogenous ubiquitin after trypsin digestion and analyzed these enriched peptides by liquid chromatography-tandem mass spectrometry (LC-MS/MS; [28–30]. The S2 ubiquitylome obtained in this way comprised more than 5800 sites present on ~1800 proteins (S2 Table). We found 7 ubiquitylation sites on MOF. Strikingly, all ubiquitylation sites were found within the chromobarrel and the HAT domains and none in the unstructured N-terminus. Instead, (Fig 2B) four of the lysines (K372, K532, K715, K798) identified in di-glycine immunoprecipitation correspond to sites ubiquitylated by MSL2 in vitro (Fig 1A).

We considered monitoring changes of MOF ubiquitylation after RNA interference against MSL2. However, upon depletion of MSL2 the levels of MOF also decrease significantly, reflecting MSL-DCC complex dissociation [16]. To investigate the contributions of MSL2 to the observed ubiquitylation pattern and at the same time boost sensitivity, we generated male (S2) and female (Kc) cell lines expressing MOF-GFP. The main qualitative difference between these two lines should be the presence of the MSL2 in S2 cells, which is not expressed in Kc cells due to the presence of the sex lethal repressor. MOF-GFP was immunopurified from whole cell extracts using the GFP-trap resin and protein species in the high molecular weight bands corresponding to ubiquitylated forms of MOF were digested in-gel and the peptides were subjected to LC-MS/MS analysis (Fig 2C and S3B and S3C Fig, S3 Table). We found MOF ubiquitylated at, K532 K715, and K798 in the structured chromobarrel and HAT domains in both male and female cells, revealing at least one other E3 ligase activity in vivo with a specificity similar to MSL2, but independent of sex. Additional ubiquitylation in female cells at K539 and K749 must not necessarily reflect female specificity, but may reflect an enhanced sensitivity due to the variability during sample preparation and analysis. No male-specific ubiquitylation of MOF was detected and neither did we find the cluster of ubiquitylation events around K153 in the unstructured N-terminus that are catalyzed by MSL2 in vitro.

### Characterization of MOF mutants in cells

The ubiquitylation within the unstructured regions of MOF, which was not detected in cells, may be present at very low levels or under certain conditions, yet still play a role for dosage compensation. This idea is particularly attractive since Akhtar and colleagues recently suggested a regulatory role for the unstructured N-terminus in regulating nucleosome binding, HAT activity and the MSL-DCC assembly [31]. Ubiquitylation of this region may prevent its folding back onto the structured domains. Likewise, the observation that all other ubiquitylation events also occurred in female cells, does not preclude a function in the context of dosage compensation, for example if the stoichiometry of MOF in its various complex contexts (MSL-DCC, NSL) was adjusted by ubiquitylating the same sites.

**Fig 3:**
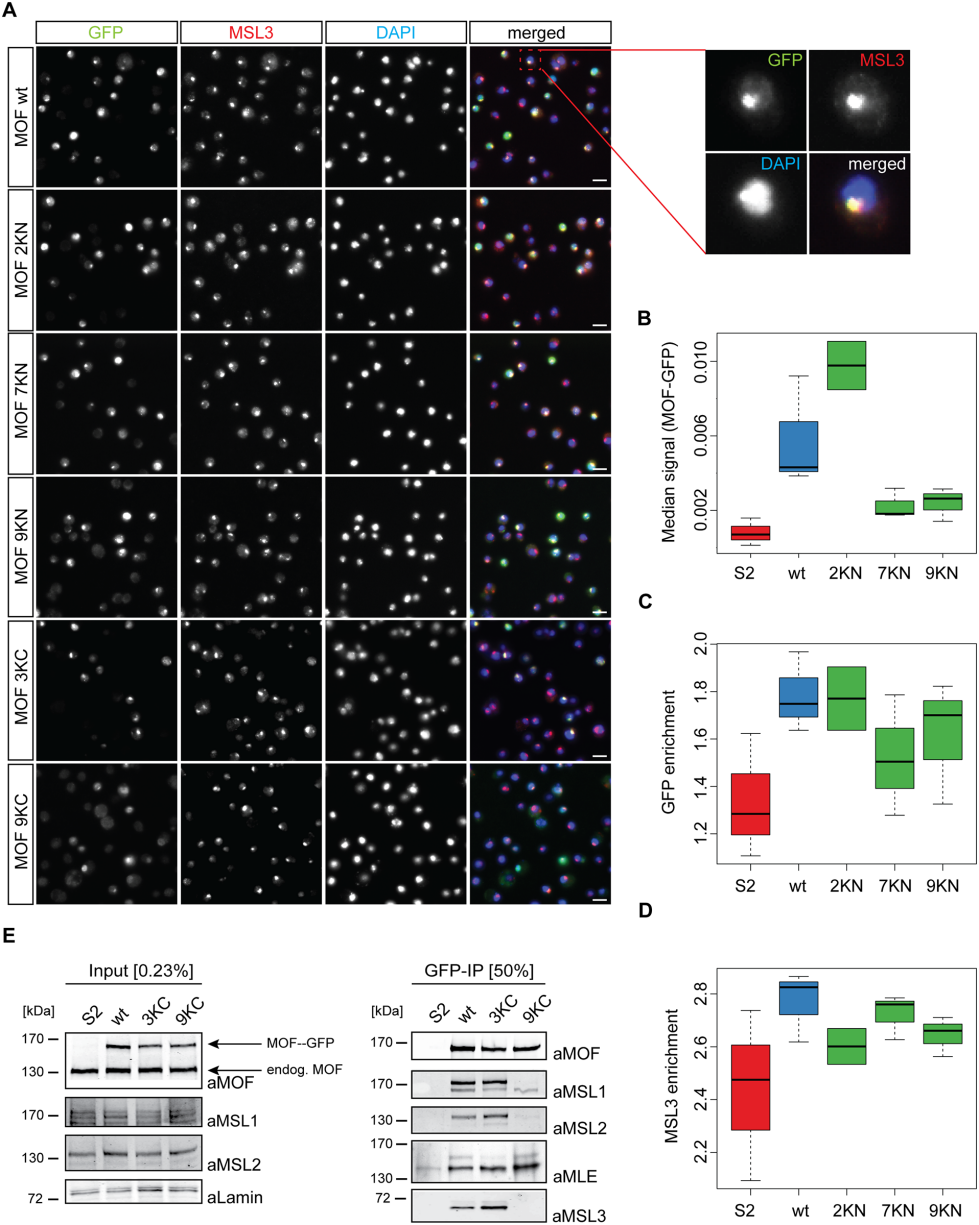
Characterization of MOF mutants in cells. (A) Nuclear localization of MOF point mutants after depletion of endogenous MOF. Stable cell lines expressing either MOF-GFP wt or MOF-GFP bearing the indicated point mutants were stained with antibodies against GFP and MSL3 as indicated. DNA was counterstained with DAPI. The GFP staining reveals the localization of the ectopic MOF, whereas MSL3 staining provides a reference for the X chromosome territory and for possible dominant negative effects of MOF mutants. The experiment was carried out in three biological replicates for MOF wt, 7KN and 9KN, 2 biological replicates are shown for 2KN. Scale bars: 10 μm. (B) Quantification of GFP immunofluorescence [data in (A)] using CellProfiler. For each cell line the median GFP signal within the nuclei (segmented on the DAPI staining) is plotted, revealing the levels of transgene expression. Non-transfected S2 cells served as GFP-negative control. The black bar indicates the median signal, the box plot presents the standard deviation. The scaling of the y-axis is logarithmic. (C) Quantification of the localization of MOF-GFP to the X-chromosome [data in (A)]. Log enrichment ratios were calculated as territorial signals computationally segmented on the MSL3 staining and the mean intensity of the nuclei (segmented on the DAPI staining). The black bar indicates average GFP enrichment for each cell line. (D) Quantification of the effect of MOF mutants on the localization of MSL3 to the X-chromosomal territories [data in (A)]. For each staining log enrichment ratios were calculated as territorial signals computationally segmented on the MSL3 staining and the mean intensity of the nuclei (segmented on the DAPI staining). The black bar indicates average MSL3 enrichment for each cell line. (E) Association of C-terminal MOF mutants with the MSL-DCC. Extracts from control cells (S2) or from cell lines stably expressing MOF-GFP-wt or the indicated mutated forms were immunoprecipitated with the GFP-trap. Western blots of input lysates or immunoprecipitates were analyzed with antibodies against MOF, MSL1, MSL2, MSL3, MLE and lamin as indicated. Protein size markers (kDa) are indicated to the left. The experiment was repeated in biological triplicates with the same outcome.

To monitor potential effects, we stably expressed transgenes coding for selected MOF derivatives (2KN, 7KN, 9KN, 3KC and 9KC) in S2 cells and monitored their nuclear localization by immunofluorescence microscopy. To promote the incorporation of exogenous MOF into the MSL-DCC we decreased the amount of endogenous MOF through RNAi interference targeting the 3’ untranslated region (UTR) of MOF mRNA (S4 Fig). MOF-wt, 2KN, 7KN, 9KN and 3KC target the X-territory similarly as assessed by colocalization with MSL3 (Fig 3A). While mutation of three C-terminal lysines (3KC) is well tolerated, mutation of 9 lysines within the HAT domain resulted in loss of GFP from the X territory. For unbiased, quantitative evaluation we employed CellProfiler [32]. We particularly focused our attention to the N-terminal mutants as these would prevent MSL2-dependent ubiquitylation in the unstructured N-terminus and chromobarrel domain. Apart from MOF 3KN, three biological replicates were quantified of each cell line. We found major differences in the median MOF-GFP signal within the nuclei (segmented on the DAPI staining) revealing major differences in expression levels for the different MOF-GFP derivatives. Compared to MOF-wt median level of 2KN was substantially higher, while mutants 7KN and 9KN were expressed particularly lowly (Fig 3B). The GFP enrichments on the X-territory followed this tendency as MOF-wt and 2KN were similarly enriched on the X, while 7KN and 9KN were less enriched (Fig 3C). At the same time MSL3 localized best to the X-territory upon expression of MOF-wt and the 7KN mutant, while its X enrichment was reduced in the presence of the 2KN and 9KN mutants (Fig 3D). The 9KC mutant appeared entirely delocalized (Fig 3A). To investigate whether this lack of targeting was due to impaired complex assembly the C-terminal MOF mutants were subject to co-immunoprecipitation using the GFP trap. MOF-3KC interacted substantially with MSL1, MSL2, MSL3 and MLE (Fig 3E). By contrast, MOF-9KC did not interact with MSL1, MSL2 and MSL3. To our surprise we found that MOF-9KC pull down substantial amounts of MLE, pointing to an ectopic interaction that so far had not been reported. In summary, we find only mild effects on MOF association with the X-chromosome upon mutating lysines in the N-terminus and CBD, while extensive mutation of C-terminal lysines results in loss of localization on the X territory as well as loss of complex association.

### Characterization of MOF mutants in flies

**Fig 4:**
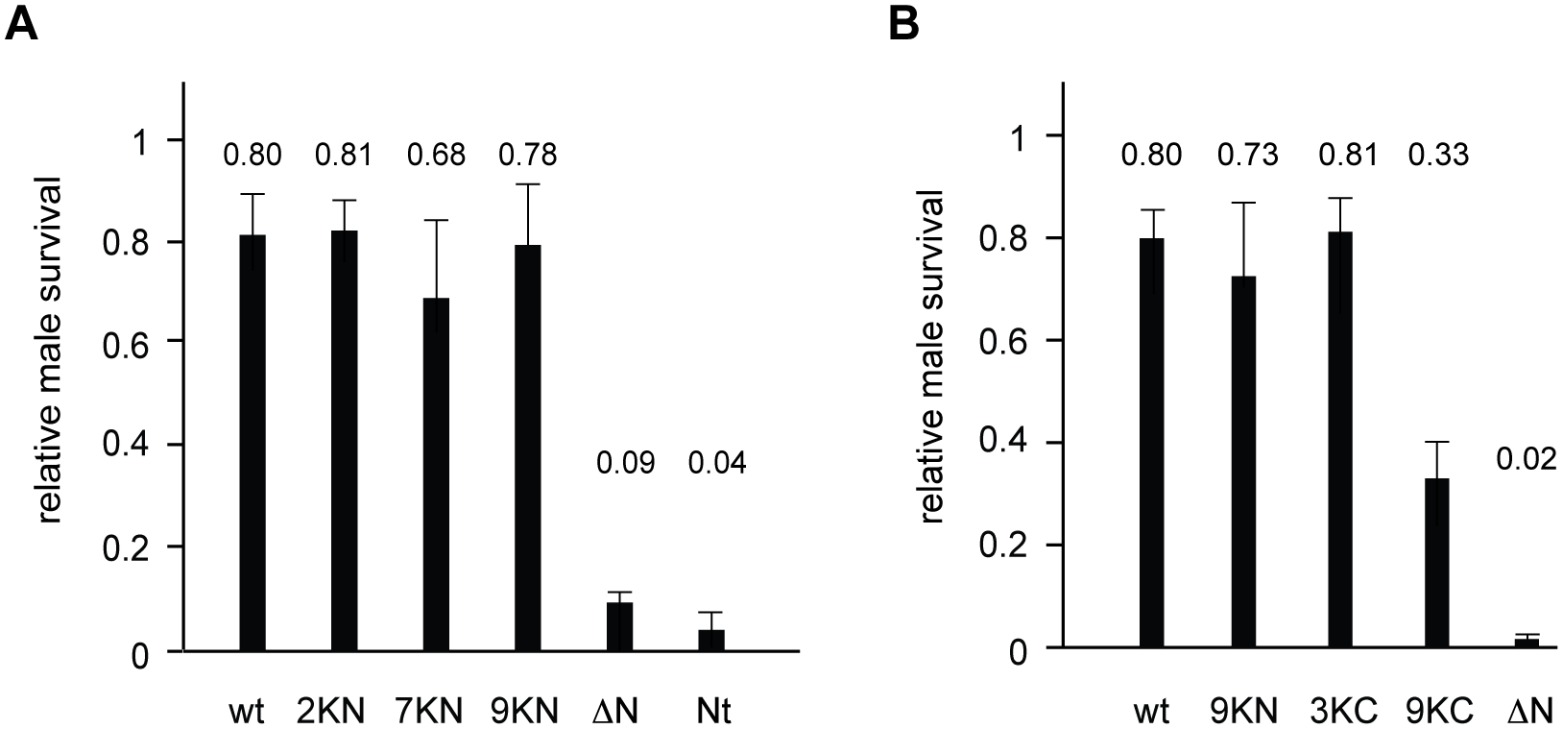
Functional viability rescue by MOF mutant enzymes. (A) Survival of MOF-deficient male flies upon expression of MOF point mutants (KN). Male survival was assayed upon expression of the indicated MOF transgenes in the *mof*^*2*^ male lethal background (see Methods). Male survival was scored as ratio *mof*^*2*^*/y* males to *mof*^*2*^*/Fm7* females resulting from the same cross (relative male survival). Error bars represent the standard error of the mean of three biological replicates. (B) Survival of *mof*^*2*^ males upon expression of MOF mutants (KC) as in (A). Error bars represent the standard error of the mean of five biological replicates.

*Drosophila* S2 cells are subject to dosage compensation, but their viability does not depend on it. As a critical test for functionality we assessed whether the MOF mutants could rescue the lethality associated with loss of endogenous MOF. Towards this end we generated transgenic flies expressing FLAG-tagged MOF-wt and MOF mutants by P element-mediated transformation. Heterozygous *mof*^*2*^ mutant flies {*mof*^*2*^*/Fm7; arm-GAL4*} that lack functional MOF protein due to a premature stop codon integrated in the *mof* coding region [33] were crossed to homozygous flies carrying MOF transgenes {*mof transgene*-UAS}. The transgenes were expressed using the UAS/GAL4 system coupled to an armadillo-GAL4 driver. Male survival was scored as ratio of *mof*^*2*^*/y* males to *mof*^*2*^*/Fm7* females resulting from the same cross. We found that the rescue efficiency depended on the number of larvae per vial. Once more than 400 flies emerged per cross the rescue efficiency decreased notably. Under those conditions, a male viability of 80% reflects maximum rescue efficiency achievable with MOF-wt (Fig 4A). MOF deletion mutants ΔN and Nt did not rescue male lethality (9% and 4%, respectively) in accordance with earlier work [31] and were used as negative control. MOF derivatives 2KN, 7KN and 9KN rescued male lethality with efficiencies comparable to MOF-wt (Fig 4A). In a second set of experiments C-terminal mutants 3KC and 9KC were investigated. To be able to compare the relative rescue efficiencies in these independent experiments the 9KN and the ΔN deletion mutants were assessed again in parallel (Fig 4B). MOF-wt rescued the *mof*^*2*^ male lethality up to 80% as before. The rescue efficiencies of MOF-9KN and -ΔN were slightly decreased compared to the previous assay (Fig 4A), but still in the expected range. Expression of MOF-3KC rescued the deficiency as well as wildtype MOF. In contrast, expression of MOF-9KC was much less able to sustain male viability with a median male survival rate of 33%. These results reveal that the mutation of 9 lysines in MOF-9KC compromises dosage compensation in flies, in agreement with its defective nuclear localization and complex formation.

### Allosteric modulation of MSL2 ubiquitylation activity

Different scenarios may explain the fact that we were unable to detect MSL2-dependent ubiquitylation of MOF in vivo. One hypothesis poses that ubiquitylation of MOF serves as a checkpoint to target wrong assemblies for degradation. Accordingly, MOF-MSL2 interactions that occur off the X chromosome may be eliminated and not be detectable, whereas chromosome-bound MOF in the context of DCC would be stable.

**Fig 5:**
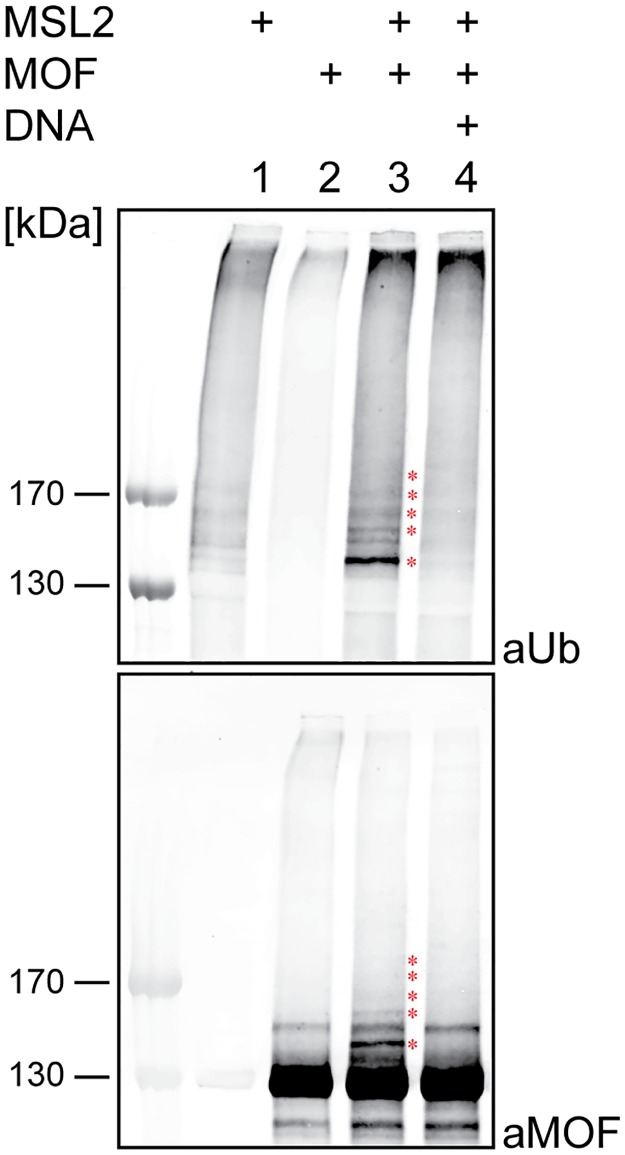
MSL2-mediated MOF ubiquitylation in vitro is inhibited by DNA. In vitro ubiquitylation assays in presence of DNA. Assays were performed as described in Fig 1C. A saturating amount of DNA (1 μM) was added to the reactions as indicated. Ubiquitylated proteins were detected using antibodies against ubiquitin (aUb) and MOF (aMOF). Protein size markers (kDa) are indicated to the left. The experiment was repeated 3 times with similar outcome.

Since MSL2 interacts directly with DNA in vitro and in vivo [11,12,34,35], we explored whether the E3 ligase activity would be modulated by DNA binding. To this end we performed in vitro ubiquitylation assays with wild type MOF as substrate in the presence of saturating amounts of 3XDBF-12-L15 DNA, a MSL-DCC binding fragment known to be a minimal sequence necessary for the recruitment of MSL2 [34]. The robust ubiquitylation of MOF by MSL2 was dramatically reduced in the presence of DNA (Fig 5). Under these conditions all MSL2 molecules are bound to DNA. We conclude that the E3 ligase activity of MSL2 can be allosterically modulated by DNA binding, a reaction that may contribute to regulated DCC assembly in vivo.

## Discussion

### Discrepancies between MOF ubiquitylation in vitro and in vivo

Two orthogonal methods, the Proximity Ligation Assay (PLA) and the direct determination of ubiquitylation sites by mass spectrometry, revealed steady-state levels of MOF ubiquitylation in *Drosophila* cell culture. The PLA signal was localized on all chromosomes and was abundant in the cytoplasm, potentially reflecting mitochondrial MOF [7]. The ubiquitylated sites in the structured C-terminal half of the protein were qualitatively similar in male or female cells, and therefore not due to the action of the male-specific MSL2 E3 ligase. It is unclear, whether this ubiquitylation reflects proteasomal degradation of MOF as part of its normal turnover, or a modification of unknown function. Strikingly, MSL2 ubiquitylated MOF at very similar sites in vitro, but in addition modified a cluster of lysines in the unstructured N-terminus (K145, K153, K170), which were not found in vivo. Our mutagenesis study revealed that in vitro MSL2 preferentially ubiquitylates lysines within the first 400 amino acids of MOF, outside its structured ‘business end’.

Despite our inability to detect ubiquitylation of MOF’s flexible N-terminus in vivo these modifications may still exist under certain circumstances. Several scenarios may be considered. First, the modified form may constitute only a small fraction of total MOF, e.g. the one that is chromosome-engaged at the X territories, and may escape detection because mass spectrometry approaches are highly biased towards abundant peptides. Conceivably, a fraction of MOF does not reside in the MSL-DCC but rather in NSL-type complexes, which regulate many promoters on all chromosomes as well as mitochondrial transcription in mammals [1,2,5,7]. MOF may be substrate of other E3 ligases (about 150 E3 ligases exist in *Drosophila* [36]), whose combined activities are likely to dominate ubiquitylation events in vivo.

Further, MSL2-dependent ubiquitylation may be a transient or rare phenomenon. If, for instance, MSL2 ubiquitylates MOF only at certain steps during the biogenesis of the MSL-DCC, only minor amounts may be present at any given time. Ubiquitylation of MOF may serve as a sensor for inappropriate conformations that target non-functional MSL assemblies for degradation. Accordingly, the N-terminus of MOF in a chromosome-bound and functional MSL-DCC would be engaged in interactions that occlude ubiquitylation. Following a similar argument, the lysines in question may be modified otherwise, for example through (auto-)acetylation and thus not be available as ubiquitylation substrate. The in vitro reaction involving only recombinant MSL2 and MOF thus reveals the potential for such ubiquitylated forms that do not accumulate in vivo. Interestingly, the N-terminus of MOF harbors a number of PEST motifs that mediate ubiquitylation for subsequent proteasomal degradation by the proteasome [37].

While non-functional assemblies may be revealed by inappropriate folding of MOF, they may also be sensed by MSL2 itself. Our observation that DNA binding significantly reduces E3 ligase activity suggests that interactions of MSL2 with MRE and PionX DNA may inhibit MOF ubiquitylation. Accordingly, MSL2 would target associated MOF for degradation if not chromatin-bound. MSL2 binds a finite number of these sequences on the X providing a mechanism to translate the number of genomic targets into appropriate numbers of MSL-DCC, a measure that may contribute to X chromosome specificity [12].

### Functional consequences of extensive lysine mutation

One surprising result of our mutagenesis study is that converting all lysines within the first 400 amino acids of MOF into arginines did not compromise MOF’s functionality in terms of interactions with other MSL subunits, acetyltransferase activity and targeting to the X chromosome. Accordingly, MOF-9KN complemented the male-lethal *mof*^*2*^ mutation equally well to wild type MOF. Evidently, ubiquitylation in this part of MOF is either not important for functionality, or its loss is effectively compensated by the organisms. By the same argument this shows that other modifications of these lysines, such as acetylation, alkylation, deamination, succinylation and SUMOylation [38] are also not vital for the fly. This neither precludes the existence of any of those modifications nor their dynamic interplay with ubiquitylation in the context of regulatory fine-tuning that our assays failed to resolve.

By contrast, mutation of 9 surface-exposed lysines in the structured C-terminal part of MOF led to impaired male viability. This mutant protein is properly folded since it shows HAT activity in vitro. Conceivably the loss of function in vivo is explained by the fact that MOF-9KC does not interact efficiently with the MSL1/2/3 module and hence is not integrated into a functional MSL-DCC. Interaction of MOF with the scaffold subunit MSL1 is required for targeting to the X chromosome (our work) and correct substrate specificity [39]. Three of the nine mutations (K539R, K541R, K545R) are predicted to be close to residues involved in interaction of MOF with MSL1, modeled according to the published interface of human MOF-MSL1 [22] It is likely that their mutation to arginine, rather than lack of ubiquitylation, perturbs binding to MSL1.

## Conclusions

We had hoped to explore the functional impact of MSL2-dependent ubiquitylation of MOF for dosage compensation. While the in vitro reaction reveals the lysines that may, in principle, be modified by MSL2, we were unable to detect MSL2-specific ubiquitylation in vivo due to the dominance of other, sex-independent ubiquitylation events. Identifying rare and transient ubiquitylation events in a subpopulation of MOF that are placed by a minor, context-dependent E3 ligase remains a technical challenge. Our study highlights the complexity of the ubiquitylation system and lists potential ubiquitylation sites as well as different ubiquitin chain linkages on MOF that can be placed by MSL2 and other E3 ligases, which can serve as starting point for future investigations with improved methodology.

## Material and methods

### Recombinant DNA

For expression of recombinant protein in Sf21 cells the cDNAs coding for MOF variants were cloned into pFastBac-1. MOF deletion mutants were obtained after restriction of pFastBac-1_MOFwt with NcoI/EcoRI for MOF-ΔN and with NcoI/SacI for MOF-Nt, respectively. Klenow Polymerase (NEB) was used to fill in 5’-overhangs and remove 3’-overhangs. The resulting DNA fragments were gel-purified and ligated using Quick T4 DNA Ligase (NEB). Lysine to arginine mutations (KN) were obtained by DNA synthesis and site directed mutagenesis. To this end two MOF sequences with either 3 (K145R, K163R, K170R) or 7 (K86R, K144R, K145R, K163R, K170R, K172R, K233R) mutations were custom-synthesized (Gene Cust) and cloned into the pBSRII vector with flanking NarI/XbalI restriction sites. The K372R and K381R mutations as well as mutations referring to MOF-3KC and -9KC were inserted by site-directed mutagenesis using primers listed in Table 1. Primers used in this study were purchased from Biomers, MWG or Sigma-Aldrich, respectively.

**Table 1:** Cloning primer for generation of MOF mutant constructs.

MOF_K372R_K381R_fw: 5’ AATAGAT**CCT**GTCGGGATTTTCGCTTATATCGAT**CCT**TTGCATC 3’
MOF_K372R_K381R_rv: 5’ GATGCAA**AGG**ATCGATATAAGCGAAAATCCCGAC**AGG**ATCTATT 3’
MOF_K532R_fw: 5’ CAGGCGGCGCTGGAG**AGG**GAGCACGAGTCCATT 3’
MOF_K532R_rv: 5’ AATGGACTCGTGCTC**CCT**CTCCAGCGCCGCCTG 3’
MOF_K706R K715R_fw: 5’ CTGTCGCGC**AGG**GAGGGCGTAATCGGTAGTCCGG**AGA**GACCGCTCTCG 3’
MOF_K706R K715R_rv: 5’ CGAGAGCGG**TCT**CTCCGGACTACCGATTACGC**CCT**CCCTGCGCGACAG 3’
MOF_K539R K541R K545R_fw: 5’ CACGAGTCCATTACG**AGG**ATC**AGG**TACATTGAT**AGG**CTGCAGTTTGGCAAC 3’
MOF_K539R K541R K545R_rv: 5’ GTTGCCAAACTGCAG**CCT**ATCAATGTA**CCT**GAT**CCT**CGTAATGGACTCGTG 3’
MOF_K694R_fw: 5’ CGTAAGGGATTTGGA**AGG**CTACTAATAGCCTTTAG 3’
MOF_K694R_rv: 5’ CTAAAGGCTATTAGTAG**CCT**TCCAAATCCCTTACG 3’
MOF_K773R K776R_fw: 5’ TCCATGAAGATGATC**AGG**TATTGG**AGG**GGCCAGAATGTCATTTGC 3’
MOF_K773R K776R_rv: 5’ GCAAATGACATTCTGGCC**CCT**CCAATA**CCT**GATCATCTTCATGGA 3’

For generation of stable cell lines MOF variants were cloned into the pHsp70-EGFP expression vector described in [16]. MOF inserts were amplified from corresponding pFastBac-1 constructs using suitable primer sets with attached AgeI/KpnI restriction sites (Table 2) and a high fidelity Pfu-DNA polymerase (Agilent Technologies). Purified PCR products and pHsp70-EGFP were digested using AgeI and KpnI restriction enzymes. Fragments were gel-purified and ligated using Quick T4 DNA Ligase (NEB).

**Table 2:** Primers for subcloning of MOF mutant constructs from pFastBac-1 to pHsp70-EGFP.

MOF_KpnI_fw: 5’ GG**GGTACC**ATGTCTGAAGCGGAGCTGG 3’
MOF-ΔN_KpnI_fw: 5’ GG**GGTACC**ATGGAGCACGACAACACTTC 3’
MOF_AgeI_rv: 5’ A**ACCGGT**GCGCCGGAATTTCCCGGAG 3’
MOF-Nt_AgeI_rv: 5’ A**ACCGGT**GCCATGGTCAAGGCATCATC 3’

For site-directed insertion of MOF expression cassettes into the fly genome the corresponding inserts were moved from the corresponding pFastBac-1 constructs to pUAST-AttB [40] using KpnI and EcoRI restriction sites.

### Recombinant proteins

MOF proteins were expressed in Sf21 cells using the Baculovirus system and purified by FLAG affinity chromatography as described in [34].

### Antibodies

MSL1, MSL2, MSL3, MOF and MLE antibodies were previously described in Gilfillan, 2006; Izzo, 2008; Prestel, 2010 [41, 42, 10]; anti-Lamin mouse monoclonal antibody (T40) was kindly provided by Prof. H. Saumweber; the anti-ubiquitin antibody was purchased from Merck Millipore (mouse, Clone FK2, #04–263); anti-GPP antibody was purchased from Roche (mouse, #11814460001) or Acris (rabbit, #TP401).

### In vitro ubiquitylation assay

Assays were carried out in 20 μl of reaction buffer (25 mM HEPES pH 7.6, 70 mM KCl, 5 mM ATP, 0.6 mM DTT) in the presence of 55 ng E1 (Boston Biochem, E-305), 263 ng E2 (Boston Biochem, E2-627), 500 ng MSL2, 2 μg His6-ubiquitin (BostonBiochem U-530), and 1 μg of substrate protein at 30°C. The reaction was stopped after 1 h by adding loading buffer. The sample was subjected to SDS-PAGE and immunoblotting.

### Fly Stocks and crosses

Fly Stocks were maintained at 18°C on standard medium. Complementation of the male-lethal *mof*^*2*^ was carried out at 25°C. Transgene expression was induced by crossing female flies of the genotype *mof*^*2*^*/Fm7a; P{armadillo-GAL4}* to male *y/w; {w*^+^ *MOF-FLAG-UAS}*. Relative male survival was scored as ratio of male *mof*^*2*^*/y; P{armadillo-GAL4}/{w*^+^ *MOF-FLAG-UAS}* to female *mof*^*2*^*/+; P{armadillo-GAL4}/{w*^+^ *MOF-FLAG-UAS}*.

### Cell culture and RNAi interference

Male cells were L2-4 cells, a clonal, tetraploid derivative of the S2 cell line that had been selected for euploidy in the lab of Patrick Heun [43]. Cells were cultivated at 26°C in Schneider’s *Drosophila* medium containing L-glutamine, and supplemented with penicillin, streptomycin and 10% (v/v) fetal calf serum (FCS). Cell density was maintained between 0.5x10^6^ and 5x10^6^ cells/ml to ensure exponential cell growth. RNA interference was performed according to standard protocols. In short, the double-stranded DNA template for production of dsRNA corresponding to SXL, MOF or GST sequences was PCR-amplified from genomic DNA or a GST cDNA with primers harboring T7 promoters. PCR products were purified using the PCR Clean-up Kit and used to transcribe dsRNA in vitro using the MEGAscript T7 kit according to the manufacturers’ specifications. dsRNA was formed by heating for 10 min at 85°C followed by slow cooling to room temperature (RT). For RNA interference 2x10^6^ exponentially growing cells were seeded to a 6-well plate in 1 ml of Schneider’s *Drosophila* medium without FCS. 10 μg of dsRNA per well was added, the reaction incubated on a shaking platform for 10 min at RT. After further incubation for 50 min at 26°C 2 ml of growth medium was added. Efficient knockdown of the target protein occurred during 6–7 days at 26°C.

### Immunofluorescence microscopy

For immunostaining 0.8×10^6^ cells were seeded onto 16 mm coverslips and allowed to attach for 1 h at RT. Cells were washed briefly with PBS and fixed in 2% (v/v) formaldehyde in PBS for 7.5 min on ice. Cells were permeabilized in PBS, 0.25% (v/v) Triton X-100, 1% (v/v) formaldehyde for additional 7.5 min on ice. Afterwards, cells were washed with PBS and blocked in PBS containing 3% (w/v) BSA for 1 h at RT. Fixed cells were incubated with suitable primary antibody diluted in blocking solution (PBS, 2% BSA, 0.01% Triton X-100, 1.2% Normal Donkey Serum) for 1 h at RT. After two wash steps cells were incubated for an additional hour with suitable secondary antibody at RT. Cells were washed again before DNA counterstaining with DAPI (1 μg/μl in PBS) for 2 min. Cells were washed twice with PBS and mounted using 10 μl Vectashield mounting medium. The coverslip was sealed to the object slide by nail polish. Immunofluorescence was detected using a Zeiss Axiovert 200 epifluorescence microscope equipped with a CDD Camera (AxioCamMR, Zeiss). Images were level-adjusted in FIJI and edited using Adobe Photoshop CS5 and Adobe Illustrator CS5.

### Proximity ligation (Duolink^®^)

0.2×10^6^ cells were seeded onto 12 mm coverslips and allowed to attach for 1 h at RT. Cells were washed briefly with PBS and subsequently fixed in 2% (v/v) formaldehyde in PBS for 7.5 min on ice. Cells were permeabilized, fixed, blocked and incubated with primary antibody incubation as above, except that primary antibody incubation was over night at 4°C in a humid chamber. Samples were further processed according to manufacturer’s guide (Duolink^®^ In Situ—Fluorescence, Sigma Aldrich).

### Ubiquitylome analysis

Confluent S2 cells were harvested from 4–5 175 cm^2^ cell culture flasks and washed twice in ice-cold PBS. The cell pellet was lysed using a modified lysis buffer (50 mM Tris-HCl/pH 7.5, 150 mM NaCl, 1 mM EDTA, 1% NP-40, 0.1% Na-deoxycholate) supplemented with protease and phosphatase inhibitors and the cysteine peptidase inhibitor N-ethylmaleimide prior to use (1:1000 Roche complete protease inhibitor cocktail, 5 mM β-glycerophosphate, 5 mM NaF, 1 mM Na-orthovanadate, 10 mM N-ethylmaleimide). Cells were incubated for 10 min on ice and centrifuged at 16,000×g for 15 minutes at 4°C. The protein concentration of the supernatant was determined using the Bradford assay. For an ubiquitylome analysis by mass spectrometry 50 mg of protein were analyzed. Proteins were precipitated in 4-fold excess of ice-cold acetone and subsequently re-dissolved in denaturation buffer (6 M urea, 2 M thiourea in 10 mM HEPES pH 8.0). Cysteines were reduced with 1 mM dithiothreitol and alkylated with 5.5 mM chloroacetamide. Proteins were digested with endoproteinase Lys-C (Wako Chemicals) and sequencing grade modified trypsin (Sigma). Protease digestion was stopped by addition of trifluoroacetic acid to 0.5% and precipitates were removed by centrifugation. Peptides were purified using reversed-phase Sep-Pak C18 cartridges (Waters) and eluted in 50% acetonitrile. Subsequently, peptides were re-dissolved in immunoprecipitation buffer (10 mM sodium phosphate, 50 mM sodium chloride in 50 mM MOPS pH 7.2). Precipitates were removed by centrifugation. Modified peptides were enriched using 40 μl of di-glycine-lysine antibody resin (Cell Signaling Technology). Peptides were incubated with the antibodies for 4 hours at 4°C on a rotation wheel. The beads were washed 3 times in ice-cold immunoprecipitation buffer followed by three washes in water. The enriched peptides were eluted with 0.15% trifluoroacetic acid in H_2_O, fractionated in 6 fractions using micro-column-based strong-cation exchange chromatography (SCX) and desalted on reversed phase C18 StageTips [44].

### Immunoprecipitation of transgenic MOF-GFP for mass spectrometry

Cells were expanded in 175 cm^2^ cell culture flasks or roller bottles. Cell pellets were washed with PBS and snap-frozen in liquid nitrogen. Cells were lysed using 5 ml RIPA extraction buffer (0.1% SDS, 0.5% deoxycholate, 0.5% NP40 (v/v), 1 mM EDTA, 50 mM Tris-Cl/pH 7.5, 150 mM NaCl) supplemented with 1 mM Protease Inhibitor, 5 mM N-ethylmaleinimid and 15 μM MG132. After 10 min incubation on ice cells were disrupted by sonication with a Branson Digital Sonifier 250D (amplitude of 15%, three 10 s pulses, 20 s pause). The lysate was incubated another 15 min on ice with occasional vortexing. The cell suspension was cleared by centrifugation at 13 krpm for 30 min at 4°C. 100 μl of equilibrated GFP-binder slurry (Chromotek) was added to the extract and incubated for 3 h on a rotating wheel at 4°C. Samples were washed in PBS / 0.1% Triton-X by inverting the tubes 20 times, followed by 3 rounds of washing with PBS / 8 M urea / 1% SDS. Finally, beads were washed with PBS / 0.1% Triton-X and pure PBS. 25% of the sample was subjected to Western blot analysis together with 0.02% of extract before immunoprecipitation (input). For mass spectrometry 75% of the sample was boiled in 5x Laemmli buffer supplemented with 1 mM DTT. After incubation with 5.5 mM 2-chloroactetamide for 45 min at RT in the dark the sample was separated by 7% SDS-PAGE. Coomassie-staining was carried out over night at RT with gentle shaking using Novex Colloidal Blue Staining Kit (Invitrogen). Proteins were digested in-gel using trypsin into peptides and extracted peptides were purified and analyzed by LC-MS/MS.

### MS analysis and peptide identification

Peptide fractions were analyzed on a quadrupole Orbitrap mass spectrometer (Q Exactive Plus, Thermo Scientific) equipped with a UHPLC system (EASY-nLC 1000, Thermo Scientific) as described [45]. Peptide samples were loaded onto C18 reversed phase columns (15 cm length, 75 μm inner diameter, 1.9 μm bead size) and eluted with a linear gradient from 8 to 40% acetonitrile containing 0.1% formic acid in 2 hours. The mass spectrometer was operated in data dependent mode, automatically switching between MS and MS2 acquisition. Survey full scan MS spectra (m/z 300–1700) were acquired in the Orbitrap. The 10 most intense ions were sequentially isolated and fragmented by higher-energy C-trap dissociation (HCD). An ion selection threshold of 5,000 was used. Peptides with unassigned charge states, as well as with charge states less than +2 were excluded from fragmentation. Fragment spectra were acquired in the Orbitrap mass analyzer. Raw data files were analyzed using MaxQuant (development version 1.5.2.8) [46]. Parent ion and MS2 spectra were searched against a database containing *Drosophila melanogaster* protein sequences obtained from the UniProtKB released in May 2016 using Andromeda search engine [47]. Spectra were searched with a mass tolerance of 6 ppm in MS mode, 20 ppm in HCD MS2 mode, strict trypsin specificity and allowing up to 3 miscleavages. Cysteine carbamidomethylation was searched as a fixed modification, whereas protein N-terminal acetylation, methionine oxidation, n-ethylmaleimide modification of cysteines (mass difference to cysteine carbamidomethylation) and di-glycine-lysine were searched as variable modifications. Site localization probabilities were determined by MaxQuant using the PTM scoring algorithm as described previously [48]. The dataset was filtered based on posterior error probability (PEP) to arrive at a false discovery rate of below 1% estimated using a target-decoy approach [49]. Di-glycine lysine modified peptides with a minimum score of 40 and delta score of 6 are reported and used for the analyses.

## Supporting information

S1 Fig Mass spectrometric fragment ion scans of the di-glycine modified peptide corresponding to lysine 153 (A), lysine 381 (B) and lysine 715 (C) in MOF. (TIF)

S2 Fig Recombinant MOF proteins employed in this study. Recombinant MOF derivatives used in in vitro assays were expressed in Sf21 cells, purified by FLAG affinity chromatography, resolved by SDS-PAGE and stained with Coomassie Brilliant blue. Protein size markers are indicated to the left (kDa). Full-length MOF (wt) migrates at 130 kDa. The deletion mutants MOF**Δ**N and MOF-Nt migrate at 55 and 80 kDa, respectively.
(TIF)

S3 Fig Detection of MOF ubiquitylation in vivo. (A) Assessment of PLA specificity to detect MOF ubiquitylation. Cell lines stably expressing MSL2-GFP were subjected to PLA assays with anti-MOF and anti-ubiquitin antibodies (first row). As controls the MOF antibody was omitted (second row) or MOF was depleted by RNA interference and both MOF and Ubiquitin antibodies were used (third row) were used. Red dots represent detected ubiquitylated MOF (PLA). X-territories are visualized in green using antibodies against MSL2-GFP (GFP). DNA was counterstained with DAPI. Scale bars represent 10 μm. (B) Mapping sites of MOF ubiquitylation by mass spectrometry. MOF-GFP was recovered from male (S2) and female (Kc) cells stably expressing MOF-GFP transgenes using the GFP-trap resin. 25% of the obtained sample was subjected to Western blot analysis. Ubiquitylated MOF species (asterisks) were detected using antibodies against MOF (aMOF, left) and ubiquitin (aUb, right) and a dual-color infrared imaging system. Low (top) or high (bottom) exposures of the Western blots are shown. Protein size markers (kDa) are indicated to the left. (C) Sample preparation for mass spectrometry. 75% of the sample mentioned in (B) was resolved by 7% SDS-PAGE and Coomassie-blue stained. The ubiquitylated protein fraction was cut from the gel as indicated by black boxes. Proteins were trypsinized and subsequently analyzed by mass spectrometry. Protein size markers (kDa) are indicated to the left.
(TIF)

S4 Fig Depletion of endogenous MOF using RNA interference. (A) S2 cell lines expressing transgenes coding for MOF-wt, and mutants 2KN, 7KN, 9KN were treated with dsRNA targeting the 3’ UTR of the endogenous MOF mRNA. Western blot analysis was performed after 7 days of RNA interference. 0.25x10^6^ cells of GST control RNAi (S2) and knockdown samples were loaded per lane and probed with antibody against lamin (aLamin) and MOF (aMOF). Protein size marker (kDa) are indicated to the left (kDa). (B) S2 cell lines expressing transgenes coding for MOF-wt, and mutants 3KC, 9KC were treated as in (A). (C) Nuclear localization of MOF-GFP upon stable expression in S2 cells. Endogenous MOF was depleted as in (A). Staining with antibodies against GFP and MSL3 as shown. DNA was counterstained with DAPI. Scale bars: 10 μm.
(TIF)

S1 Table All di-glycine lysine sites identified on MOF and ubiquitin in MSL2 in vitro ubiquitylation assays. Sites identified in 3 biological replicates are depicted in grey.
(XLSX)

S2 Table All di-glycine lysine sites identified in vivo by ubiquitin remnant profiling from Drosophila S2 cells. (XLSX)

S3 Table All di-glycine lysine sites identified on overexpressed MOF-GFP in male and female Drosophila cells. (XLSX)
