# Supplementary figures and images for: Ubiquitylation of the acetyltransferase MOF in Drosophila melanogaster
Source: PLoS One. 2017 May 16;12(5):e0177408. doi: 10.1371/journal.pone.0177408 (PMC5433716; doi:10.1371/journal.pone.0177408)

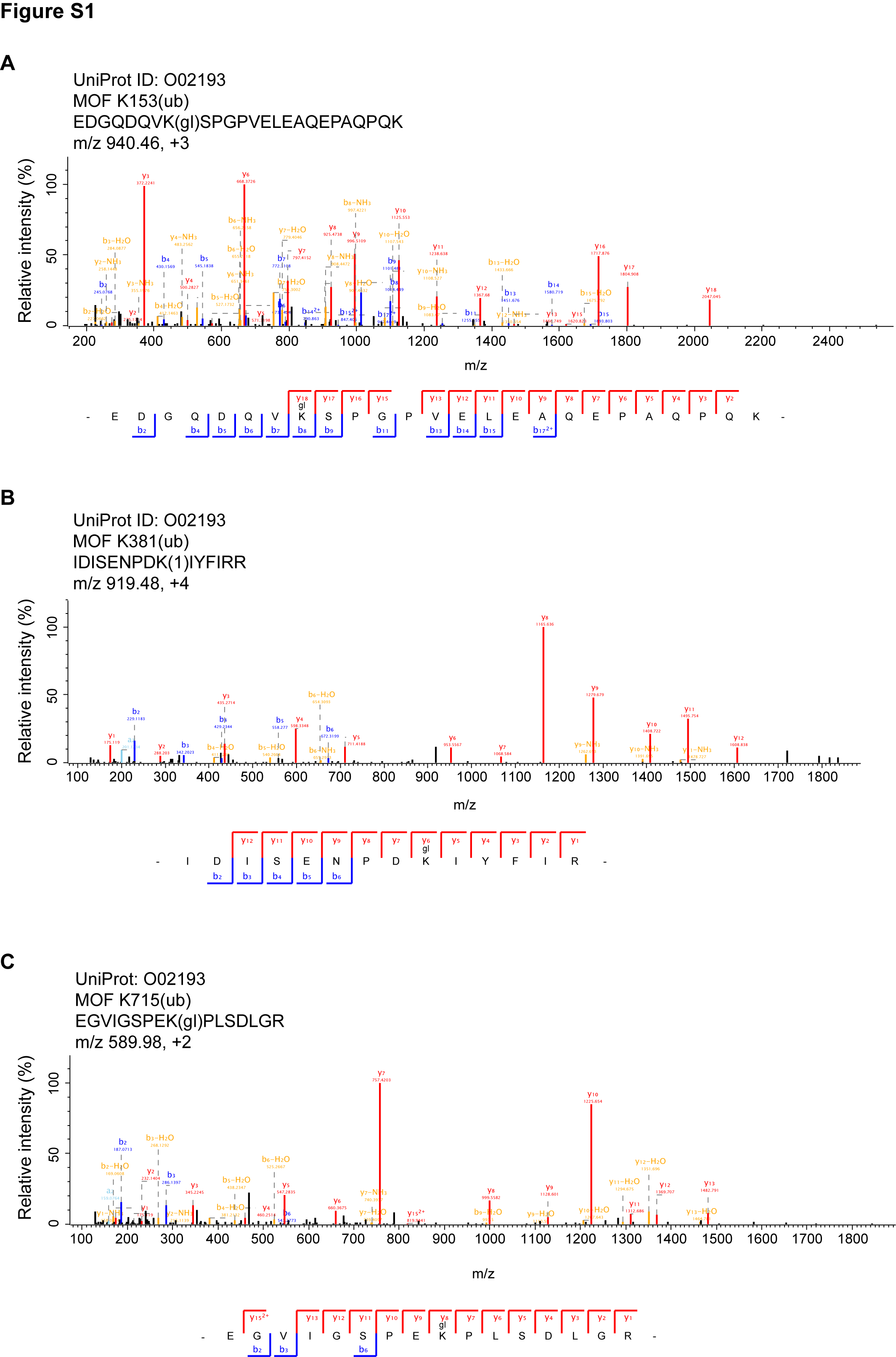

Supplement: S1 Fig — (TIF) [file pone.0177408.s001.tif]

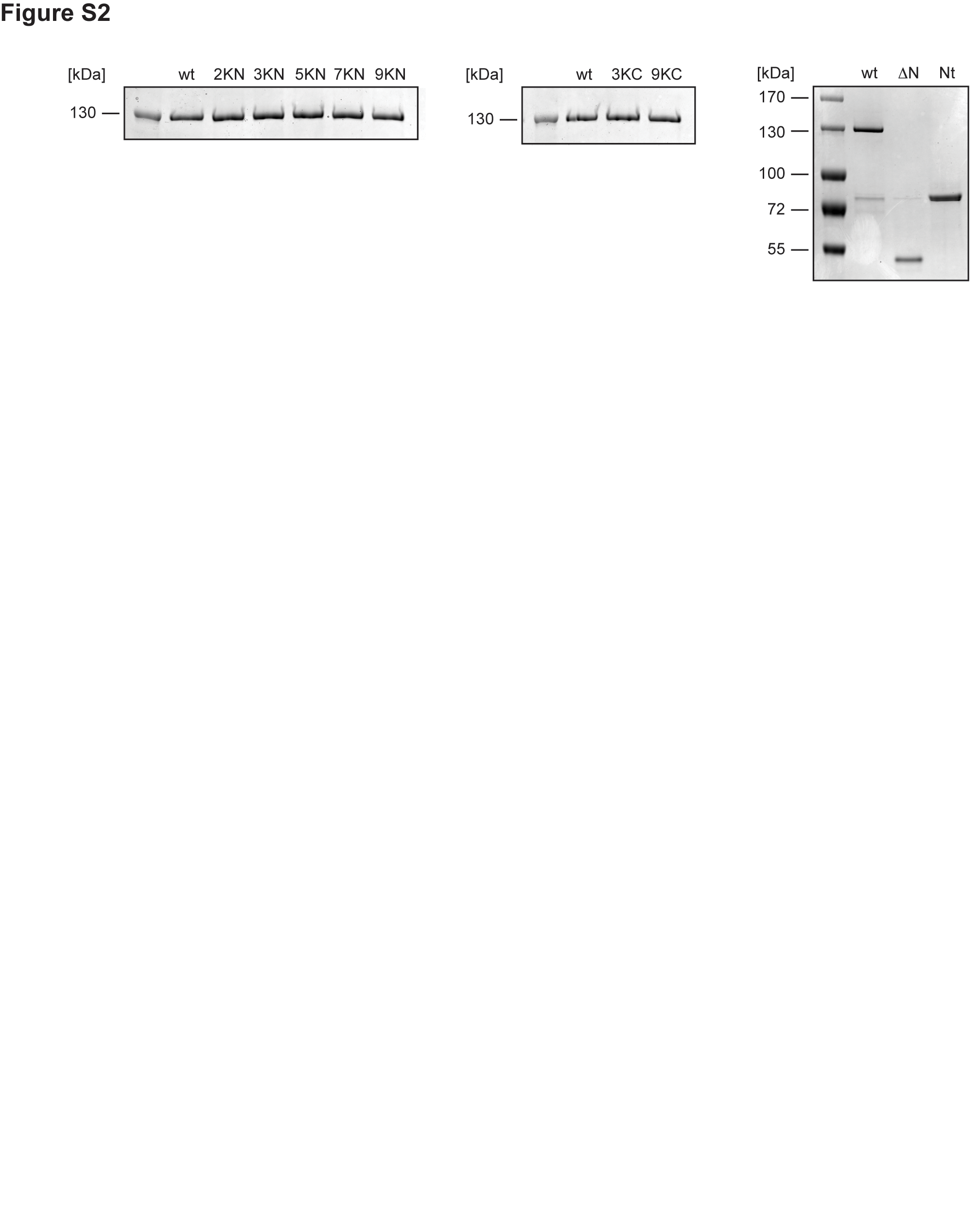

Supplement: S2 Fig — Recombinant MOF derivatives used in in vitro assays were expressed in Sf21 cells, purified by FLAG affinity chromatography, resolved by SDS-PAGE and stained with Coomassie Brilliant blue. Protein size markers are indicated to the left (kDa). Full-length MOF (wt) migrates at 130 kDa. The deletion mutants MOFΔN and MOF-Nt migrate at 55 and 80 kDa, respectively. (TIF) [file pone.0177408.s002.tif]

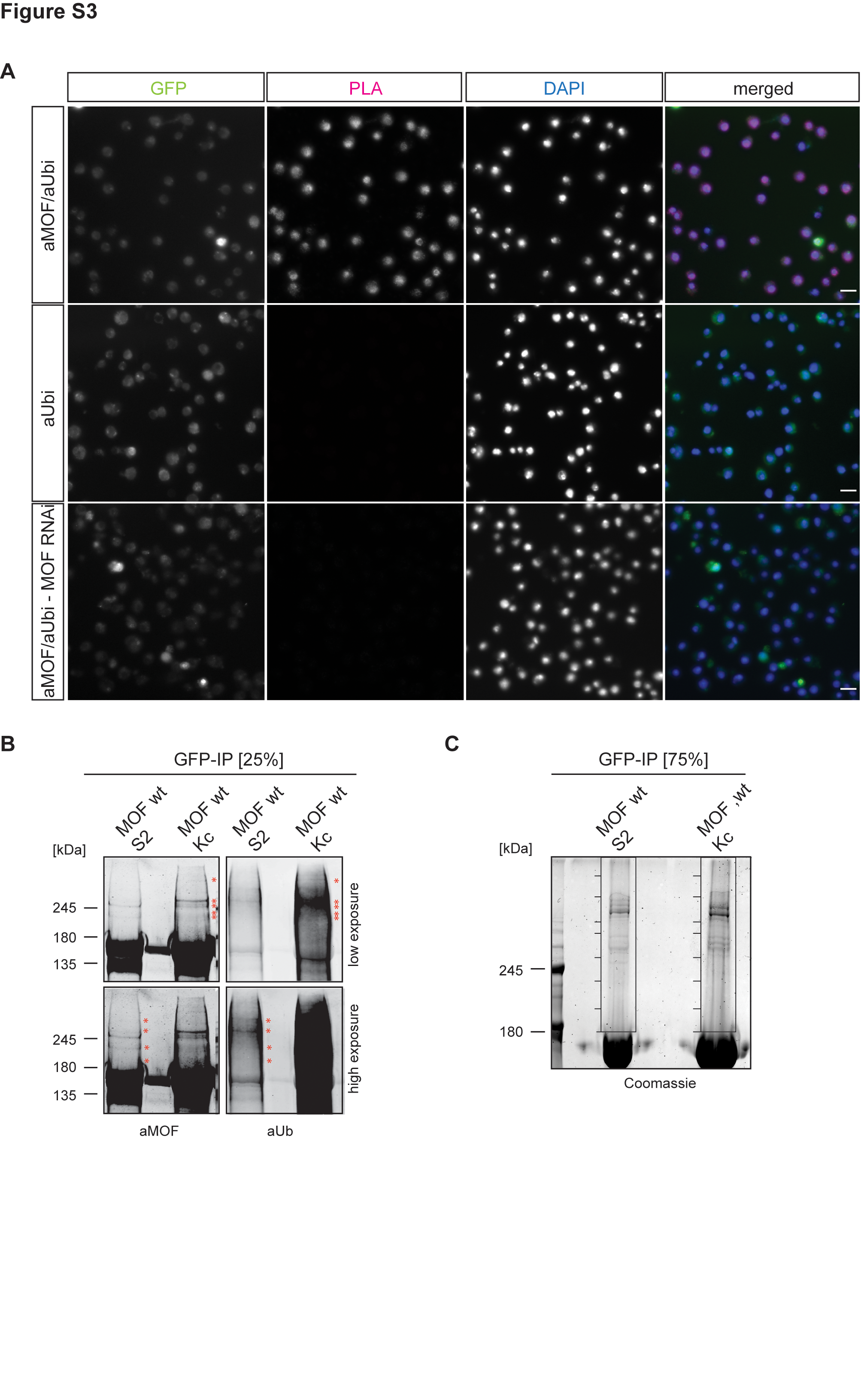

Supplement: S3 Fig — (A) Assessment of PLA specificity to detect MOF ubiquitylation. Cell lines stably expressing MSL2-GFP were subjected to PLA assays with anti-MOF and anti-ubiquitin antibodies (first row). As controls the MOF antibody was omitted (second row) or MOF was depleted by RNA interference and both MOF and Ubiquitin antibodies were used (third row) were used. Red dots represent detected ubiquitylated MOF (PLA). X-territories are visualized in green using antibodies against MSL2-GFP (GFP). DNA was counterstained with DAPI. Scale bars represent 10 μm. (B) Mapping sites of MOF ubiquitylation by mass spectrometry. MOF-GFP was recovered from male (S2) and female (Kc) cells stably expressing MOF-GFP transgenes using the GFP-trap resin. 25% of the obtained sample was subjected to Western blot analysis. Ubiquitylated MOF species (asterisks) were detected using antibodies against MOF (aMOF, left) and ubiquitin (aUb, right) and a dual-color infrared imaging system. Low (top) or high (bottom) exposures of the Western blots are shown. Protein size markers (kDa) are indicated to the left. (C) Sample preparation for mass spectrometry. 75% of the sample mentioned in (B) was resolved by 7% SDS-PAGE and Coomassie-blue stained. The ubiquitylated protein fraction was cut from the gel as indicated by black boxes. Proteins were trypsinized and subsequently analyzed by mass spectrometry. Protein size markers (kDa) are indicated to the left. (TIF) [file pone.0177408.s003.tif]

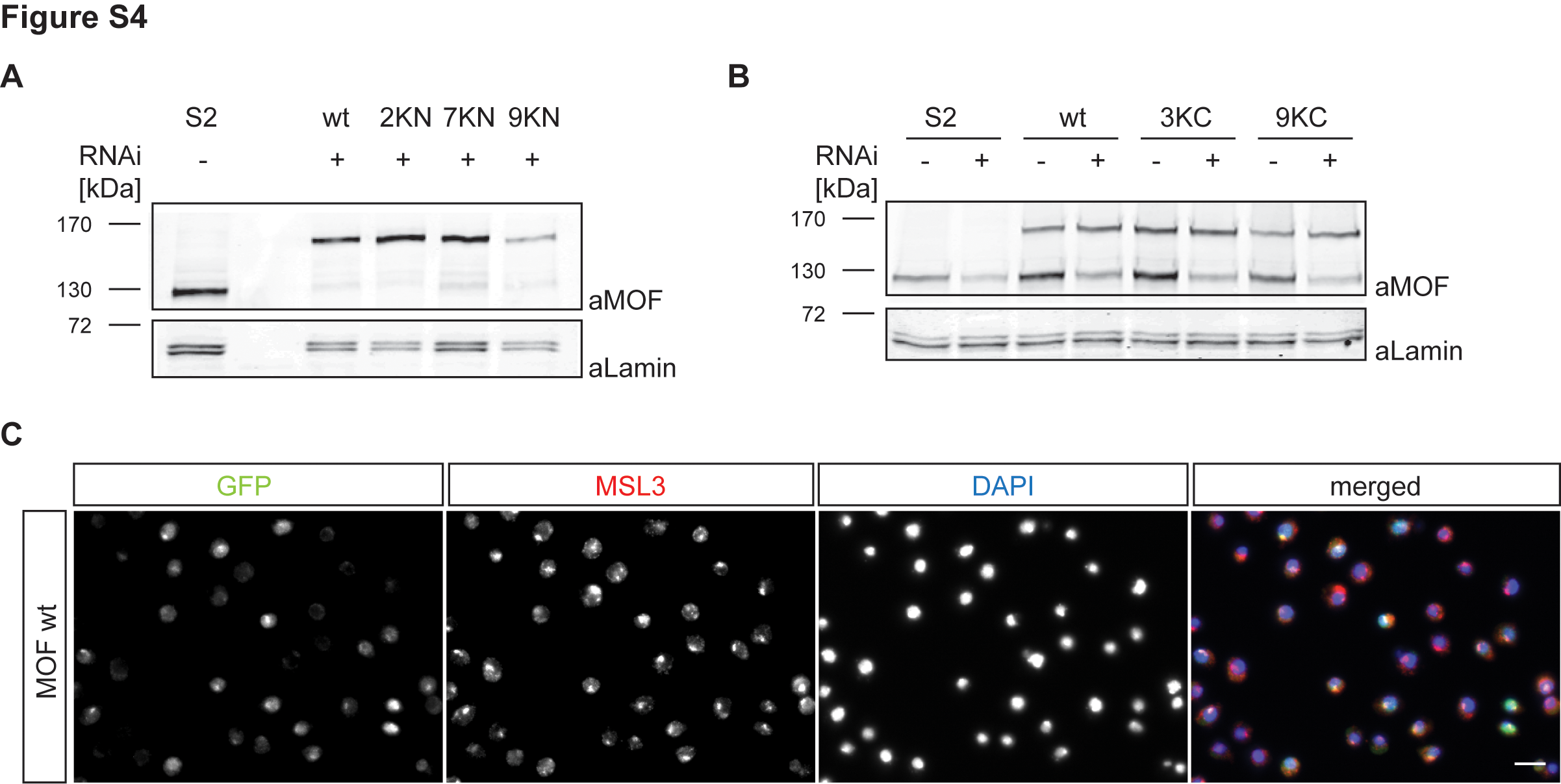

Supplement: S4 Fig — (A) S2 cell lines expressing transgenes coding for MOF-wt, and mutants 2KN, 7KN, 9KN were treated with dsRNA targeting the 3’ UTR of the endogenous MOF mRNA. Western blot analysis was performed after 7 days of RNA interference. 0.25x10^6^ cells of GST control RNAi (S2) and knockdown samples were loaded per lane and probed with antibody against lamin (aLamin) and MOF (aMOF). Protein size marker (kDa) are indicated to the left (kDa). (B) S2 cell lines expressing transgenes coding for MOF-wt, and mutants 3KC, 9KC were treated as in (A). (C) Nuclear localization of MOF-GFP upon stable expression in S2 cells. Endogenous MOF was depleted as in (A). Staining with antibodies against GFP and MSL3 as shown. DNA was counterstained with DAPI. Scale bars: 10 μm. (TIF) [file pone.0177408.s004.tif]
